# Acute Hemodynamic Effects of Simultaneous and Sequential Multi-Point Pacing in Heart Failure Patients With an Expected Higher Rate of Sub-response to Cardiac Resynchronization Therapy: Results of Multicenter SYNSEQ Study

**DOI:** 10.3389/fcvm.2022.901267

**Published:** 2022-05-12

**Authors:** Maciej Sterliński, Joanna Zakrzewska-Koperska, Aleksander Maciąg, Adam Sokal, Joaquin Osca-Asensi, Lingwei Wang, Vasiliki Spyropoulou, Baerbel Maus, Francesca Lemme, Osita Okafor, Berthold Stegemann, Richard Cornelussen, Francisco Leyva

**Affiliations:** ^1^First Department of Arrhythmia, National Institute of Cardiology, Warsaw, Poland; ^2^Second Department of Arrhythmia, National Institute of Cardiology, Warsaw, Poland; ^3^Department of Cardiology, Congenital Heart Diseases and Electrotherapy, Silesian Center of Heart Disease, Zabrze, Poland; ^4^Cardiology Department, University and Polytechnic Hospital la Fe, Valencia, Spain; ^5^Section of Arrhythmias, Department of Cardiology, Clinical Sciences, Skåne University Hospital, Lund University, Lund, Sweden; ^6^Bakken Research Center, Medtronic plc, Maastricht, Netherlands; ^7^Queen Elisabeth Hospital, Birmingham University, Birmingham, United Kingdom; ^8^Aston Medical School, Aston Medical Research Institute, Aston University, Birmingham, United Kingdom

**Keywords:** heart failure, biventricular pacing, quadripolar lead for left ventricle pacing, multipoint pacing, acute hemodynamic effect, cardiac resynchronization therapy

## Abstract

The aim of the SYNSEQ (Left Ventricular Synchronous vs. Sequential MultiSpot Pacing for CRT) study was to evaluate the acute hemodynamic response (AHR) of simultaneous (3P-MPP _syn_) or sequential (3P-MPP _seq_) multi-3-point-left-ventricular (LV) pacing vs. single point pacing (SPP) in a group of patients at risk of a suboptimal response to cardiac resynchronization therapy (CRT). Twenty five patients with myocardial scar or QRS ≤ 150 or the absence of LBBB (age: 66 ± 12 years, QRS: 159 ± 12 ms, NYHA class II/III, LVEF ≤ 35%) underwent acute hemodynamic assessment by LV + dP/dt_max_ with a variety of LV pacing configurations at an optimized AV delay. The change in LV + dP/dt _max_ (%ΔLV + dP/dt _max_) with 3P-MPP _syn_ (15.6%, 95% CI: 8.8%-22.5%) was neither statistically significantly different to 3P-MPP _seq_ (11.8%, 95% CI: 7.6-16.0%) nor to SPP _basal_ (11.5%, 95% CI:7.1-15.9%) or SPP _mid_ (12.2%, 95% CI:7.9-16.5%), but higher than SPP _apical_ (10.6%, 95% CI:5.3-15.9%, *p* = 0.03). AHR (defined as a %ΔLV + dP/dt _max_ ≥ 10%) varied between pacing configurations: 36% (9/25) for SPP _apical_, 44% (11/25) for SPP _basal_, 54% (13/24) for SPP _mid_, 56% (14/25) for 3P-MPP _syn_ and 48% (11/23) for 3P-MPP _seq.Fifteen_ patients (15/25, 60%) had an AHR in at least one pacing configuration. AHR was observed in 10/13 (77%) patients with a LBBB but only in 5/12 (42%) patients with a non-LBBB (*p* = 0.11). To conclude, simultaneous or sequential multipoint pacing compared to single point pacing did not improve the acute hemodynamic effect in a suboptimal CRT response population.

**Clinical Trial Registration:**

ClinicalTrials.gov, identifier: NCT02914457.

## Introduction

Cardiac resynchronization therapy (CRT) has transformed the treatment of patients with heart failure, impaired left ventricular (LV) function and a wide QRS complex (1). It is well accepted, however, that the response to CRT delivered using bipolar and unipolar leads is variable. Quadripolar LV leads are associated with higher implant success rates, lower rates of re-interventions for LV lead displacement or phrenic nerve stimulation (2–4) and better clinical outcomes (3, 4).

Intuitively, the wide LV activation front provided by simultaneous, multipoint pacing (MPP_syn_) could achieve a more rapid and uniform LV activation than single point pacing (SPP). A better acute hemodynamic response (AHR) to CRT with MPP compared to SPP has been reported by some studies (5, 6), but not others (7, 8). It has also been shown that MPP confers a better LV reverse remodeling response to CRT compared to SPP (9). With respect to clinical outcomes, some studies showed a superiority of MPP over SPP (2), but this was not supported by a recent randomized, controlled trial (10). Physiologically, sequential MPP from apex to base (MPP_seq_) could also provide a physiological pattern of LV activation (11, 12). In this respect, a favorable response to CRT delivered using apical LV pacing is consistent with the notion that CRT, delivered using LV sequential activation from apex to base may be more physiological and therefore, more advantageous (13–15).

Response to CRT still raises many questions and there is a large population of subjects in which CRT brings moderate or even no benefit (16). Ischemic cardiomyopathy (17, 18), a relatively narrow QRS complex (19) and an non-LBBB morphology are associated with a higher risk of incomplete or poor/absent clinical improvement due to CRT (“sub-response”) (20). In this experimental, interventional study, we compare the acute hemodynamic effect in presumed sub-responders to CRT delivered using SPP as well as 3-point, simultaneous (3P-MPP_syn_) or sequential (3P-MPP_seq_) MPP pacing. Recent data show that acute hemodynamic response measured by LV dP/dtmax is correlated with better clinical outcome and reverse remodeling, expressed as reduction of LVESV and LVEF improvement (21). Therefore, our work is part of the search for more effective resynchronizing stimulation techniques in a “sub-response” group. At the same time it offers new perspectives on this topic.

## Methods

### Study Design

The SYNSEQ (Left Ventricular Synchronous versus Sequential MultiSpot Pacing for CRT) study (NCT02914457) was an acute hemodynamic study with prospective enrolment, conducted across five European centers. All patients provided written informed consent. The study was approved by the Local Ethics Committees and complied with the Declaration of Helsinki.

### Study Population

LBBB morphology on ECG was defined using the Strauss criteria (22). Patients diagnosed with LBBB with QRS > 150 ms together with absence of scar or patients having pure RBBB were not allowed in the study. Deviations from the above morphology in more than two surface ECG leads were classified as non**-**LBBB. ECG morphology was assessed independently by two blinded investigators. The etiology of heart failure was confirmed on basis of clinical history, and the echocardiographic examination. In addition, transmural/subendocardial myocardial scar was accessed by late-gadolinium enhancement cardiac magnetic resonance (23). All inclusion and exclusion criteria are listed in Table 1. This specific population was chosen based upon the (a) the relatively low-response acutely and chronic and therefore represent an opportunity for an experimental LV stimulation model, and (b) that typical-LBBB patients with relatively wide QRS and no scar do in general respond very well to conventional CRT-therapy.

**Table 1:** Study inclusion and exclusion criteria.

**Inclusion criteria**	**Exclusion criteria**
CRT indication according to the present ESC/AHA guidelines and: a. Presence of myocardial scar or b. QRS duration ≤ 150 ms or c. Non-LBBB • Sinus rhythm • Oral optimal medical treatment • Voluntary participation in the study and signing of informed consent • ≥18 year old	• Permanent atrial fibrillation/flutter or other supraventricular tachycardia • Pure right bundle branch block (with no additional left ventricular conduction delays) • Myocardial infarction or valve surgery within 40 or, respectively, 90 days prior to enrollment • Severe aortic stenosis with area <1.0 cm^2^ or significant valve disease expected to be operated within the study period • Mechanical heart valves • Congenital heart disease • HT or active on the transplantation list • LVAD • Severe renal disease (up to physician's discretion) • Continuous or uninterrupted infusion (inotropic) therapy for heart failure (≥2 stable infusions per week) • Pregnant or breastfeeding woman • Participation in another study that confound the results of this study, without documented pre-approval.

*LBBB, left bundle branch block; non-LBBB, deviations from the LBBB morphology, according the Strauss criteria, in more than two surface ECG leads; MRI, Magnetic Resonance Imaging; LVAD, left ventricular assist device; HT, heart transplant*.

### Lead Implantation

**Figure 1:**
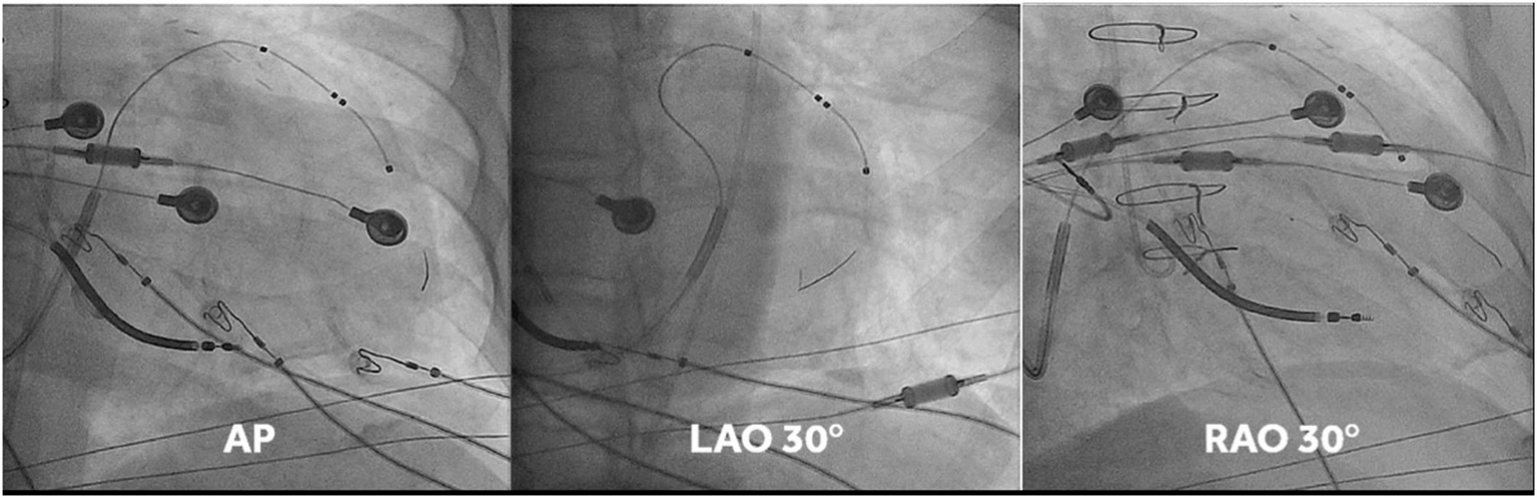
Fluoro-images at AP, LAO 30° and RAO 30° displaying position of the different CRT leads. Note that in this case the vision wire administered through the lumen of the quadripolar was used to obtain true apical position. MPP was delivered on the vision wire, and on the distal and most proximal electrodes of the short bipole of the quadripolar lead.

This was undertaken using standard transvenous techniques with cephalic, axillary or subclavian access. Right atrial and right ventricular leads were first deployed into typical locations (preferred right atrium appendage if possibly and right ventricular apex or low septum, respectively), followed by deployment of a quadripolar LV lead within the vein chosen by implanters, who were instructed to deploy the LV lead tip as apical as possible within the vein of choice (an example of lead placement is shown in Figure 1). If the apex could not be reached with a transvenous LV lead, a 0.14” pacing wire (VisionWire, Biotronik, Berlin) was used for apical pacing. Apical position was defined by 30° RAO fluoroscopy as the lowest quartile in the longitudinal direction and was achieved in 100% of the patients. Acceptable LV lead position was either lateral or posterolateral (Figure 1).

### Lead Positions

Anteroposterior, left anterior oblique (30°) and right anterior oblique (30°) fluoroscopic views were used to localize lead positions, as previously described (12). Briefly, the position of pacing poles was determined by measuring the distance from the coronary sinus to the apex, using 30° right anterior oblique fluoroscopic view. The circumferential position over the LV free wall was determined using the o'clock method, assuming that the anterior interventricular vein was at a 12 o'clock position and the inferior vein at a 6 o'clock position. Thus, the LV pacing pole position (basal, mid, and apical) refers to the subtended myocardial segments, rather than the position of the pacing poles on the lead.

### Pacing Protocol

The acute hemodynamic study was undertaken during implantation of a CRT device. The CRT implantation was performed as per standard practice after completion of the acute study. Four external pacemakers (Medtronic Model 5388, Medtronic, MN), synchronized by a central master pacemaker (Analyzer Medtronic 2290, Medtronic, MN) and a custom-made switch box, were used for each pacing site to ensure capture. The atrial channel of the central master was used for right atrial pacing. Throughout the acute study, cardiac electrograms, surface ECGs, invasive arterial blood pressure (femoral artery) and LV pressure (MicroCath Millar instruments, TX, USA**)** were acquired with a 32-channel recording system (Porti TMSi, Oldenzaal, Netherlands) and recorded on a laptop computer using customized software. Beat-to-beat raw signals were visualized and checked in real time to ensure appropriate signal quality and to confirm capture. Experimental lead configurations and atrioventricular (AV) delay settings were digitally annotated for off-line analysis.

The reference for calculation of %ΔLV + dP/dt_max_ was AAI pacing 10 bpm above the intrinsic rate. For AV optimization, LV + dP/dt_max_ was measured at five different AV delays, namely the AV delay determined by the CardioSync algorithm (Medtronic, MN) and AV delays of ±30 and ±60 ms around this AV delay. All measurements were repeated 4 times over 20 beats for each pacing configuration and AV delay, interspersed with AAI pacing, to minimize sampling error (24). The inter-ventricular (VV) pacing delay was set to zero for all configurations except for the 3-P MPP_seq_ (VV-delay = 20ms between LV_apex_ and LV_mid_ and between LV_mid_ and LV_basal_). The tested LV pacing configurations were RV and SPP_apex_, RV and SPP_mid_, RV and SPP_basal_, RV and 3P-MPP_syn_, RV and MPP_seq_. For analysis, up to eight beats prior and eight beats immediately after each pacing change from a pacing configuration to AAI pacing were used to calculate percentage change in LV + dP/dt_max_.

### Hemodynamic Endpoint

Acute hemodynamic effect (AHE) was assessed as the percentage change in LV + dP/dt_max_ (%ΔLV + dP/dt_max_) from pacing on to pacing off (AAI). The acute hemodynamic response (AHR) was defined as ≥10% increase in the acute hemodynamic effect (%ΔLV + dP/dt_max_).

### Data Analysis

Beat-to-beat LV intraventricular pressure, 12 lead surface ECG and endocardial (RA, RV, and LV) electrograms were acquired simultaneously using a 32-channel physiological recording system (Porti, TMSi, Twente, The Netherlands). Data analysis was undertaken offline. The Raschlab v0.3.0 software package (Raphael Schneider, Medtronic Inc.) was used for data review and annotation. Non-captured beats and ventricular ectopic beats plus the subsequent two beats were identified visually and excluded from further analyses. The dataset was then converted to Matlab (The Mathworks Inc., Massachusetts) compatible format for further analysis.

The AHE for each configuration was calculated with the median LV + dP/dt_max_ for up to eight cardiac beats before and after the experimental transition from pacing on to pacing off. We then calculated ΔLV + dP/dt_max_ for each of the eight transitions.

The paced QRS duration was measured from the ventricular pacing spike to the end of the QRS complex in surface ECGs. The Q-LV interval was defined as the interval from the onset of the intrinsic QRS on the surface ECG to the first large positive or negative peak of the LV electrogram. Q-LV-timing data are expressed as Q-LV/QRS. The electrical delay from RV or LV pacing spike to the different LV activations was also measured.

### Statistical Analysis

Statistical analysis was performed using SAS 9.4 (SAS Institute, Cary, NC) and R (versions up to 3.6.1). Primary objectives: For comparison between pacing configurations, the following approach was performed. Firstly, the maximal average %ΔLV + dP/dt_max_ was calculated for each subject and each configuration by a regression analysis constructing a quadratic curve through all AV-delays (25, 26). Secondly, two-sided (except for non-inferiority which is one-sided per definition) weighted paired *t*-tests were performed to compare the pacing configurations to each other. Subjects were inversely weighted per comparison based on the model estimated variability of their maximal average %ΔLV + dP/dt_max_ for the compared configurations. Sensitivity analyses were performed comparing analysis results between a non-parametric Wilcoxon signed-rank test, unweighted *t*-test and weighted *t*-test. Two-tailed *p*-values smaller than 0.05 and one-tailed *p*-values smaller than 0.025 were considered significant. *P*-values are presented as two-sided unless indicated otherwise. For the comparison between 3P-MPP_seq_ and 3P-MPP_syn_, non-inferiority testing was performed using a margin of −3% and a significance level of 0.025. If non-inferiority testing was significant, a test for superiority at a significance level of 0.05 was performed. Categorical variables were compared using Fisher's exact test. Binomial sample proportions were compared to expected percentages using a one-sided Wald test to see whether one configuration was more often the best one than would be expected by chance. Secondary objectives: Linear multiple regression analysis was used to assess correlation between %ΔLV + dP/dt_max_ and Q-LV/QRS ratio or ΔQRS duration.

Continuous variables are expressed as mean ± SD (unless indicated otherwise). No correction for multiple testing was performed because of the exploratory nature of this study.

## Results

**Figure 2:**
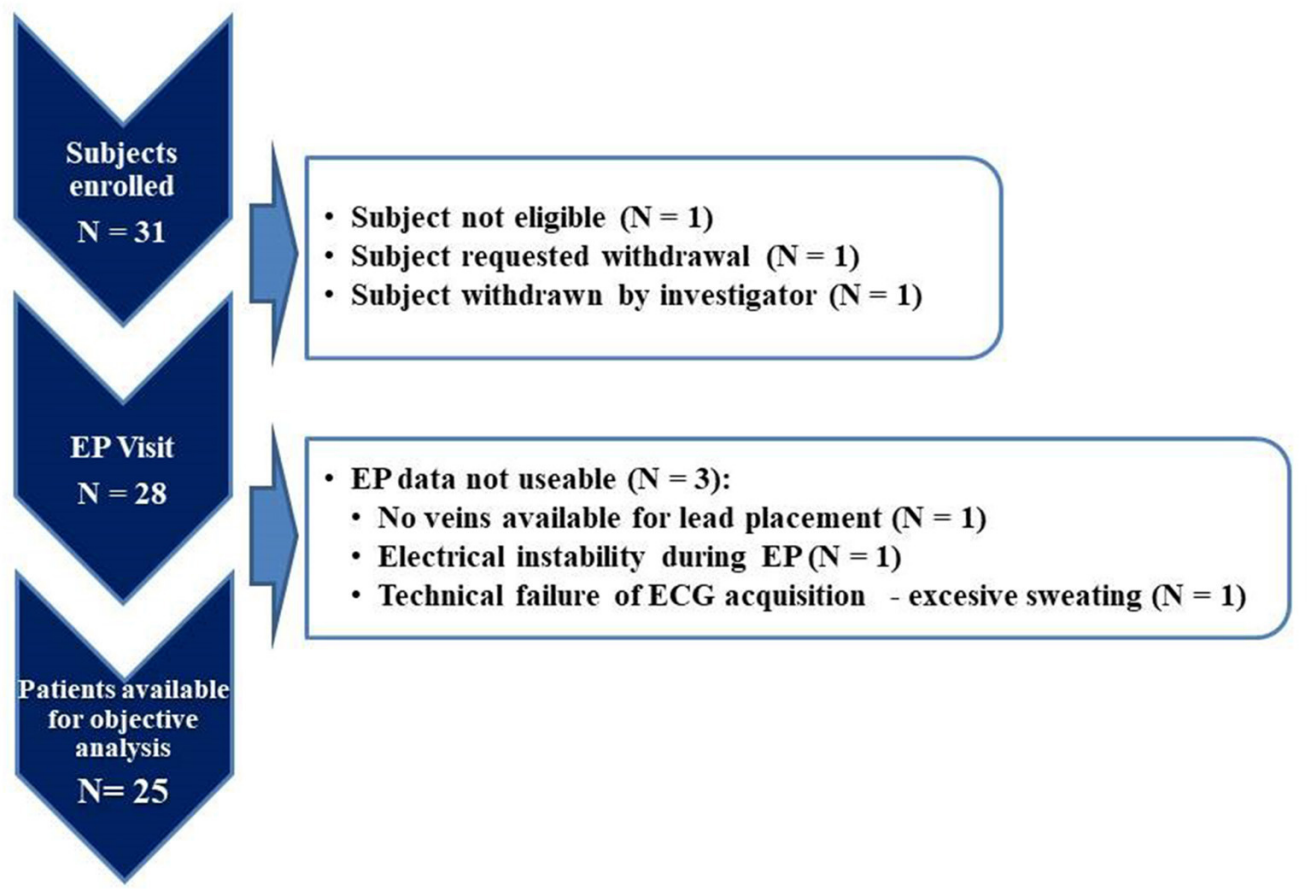
SYNSEQ study flowchart.

Thirty-one patients were enrolled in the study. Complete datasets were available for analysis for 25 patients (study flowchart is shown in Figure 2). For comparison of typical LBBB vs. non-LBBB the groups size was only 13 and 12 patients, respectively, indicating only a proof-of-principle (see also Limitations in the Discussion).

### Baseline Characteristics of Patients With Complete Datasets

There were 25 subjects, (age: 66 ± 12 yrs [mean ± SD], 80% male), 12 of whom (12/25, 48%) showed no typical LBBB pattern on ECG. Twenty patients presented with myocardial scar (20/25, 80%), and 10 had a QRS-duration ≤ 150 (10/25, 40%). Patients received maximally tolerated pharmacological therapy for heart failure prior to the CRT implant. Patients' demographics are summarized in Table 2. No arrhythmias were induced during any of the pacing protocols. Data on the duration of the electrophysiological measurements determined by the protocol are included in Supplementary Table 1.

**Table 2:** Characteristics of the study group.

**Subjects characteristics**	**All** **(*N* = 25)**	**LBBB** **(*N* = 13)**	**Non-LBBB** **(*N* = 12)**
Sex (male), *n* (%)	20 (80.0%)	9 (69.2%)	11 (91.7%)
Age, years	66.2 (11.9)	64.8 (14.1)	67.7 (9.5)
NYHA class II, *n* (%)	12 (48.0%)	7 (53.8%)	5 (41.7%)
NYHA class III, *n* (%)	13 (52.0%)	6 (46.2%)	7 (58.3%)
LVEF, %	26.0 (5.0)	27.4 (5.2)	24.4 (4.4)
Myocardial infarction, *n* (%)	17 (68.0%)	7 (53.8%)	10 (83.3%)
Scar (LGE), *n* (%)^*^	20 (80.0%)	11 (84.6%)	9 (75.0%)
**Comorbidity**, ***n*** **(%)**			
Diabetes	9 (36.0%)	3 (23.1%)	6 (50.0%)
Hypertension	17 (68.0%)	7 (53.8%)	10 (83.3%)
CABG	6 (24.0%)	2 (15.4%)	4 (33.3%)
**ECG variables**			
PR interval, ms	190.2 (32.9)	191.7 (37.4)	188.5 (28.8)
QRS duration, ms	158.7 (11.9)	160.0 (9.8)	157.3 (14.2)
**Medications**, ***n*** **(%)**			
Diuretics	20 (80.0%)	10 (76.9%)	10 (83.3%)
ACEIs/ARBs	22 (88.0%)	11 (84.6%)	11 (91.7%)
Beta-blockers	23 (92.0%)	11 (84.6%)	12 (100.0%)
Aldosterone antagonists	24 (96.0%)	12 (92.3%)	12 (100.0%)

* *Two Non-LBBB subjects did not have MRI scan performed*.

### Effect of Simultaneous and Sequential Pacing Configurations

**Figure 3:**
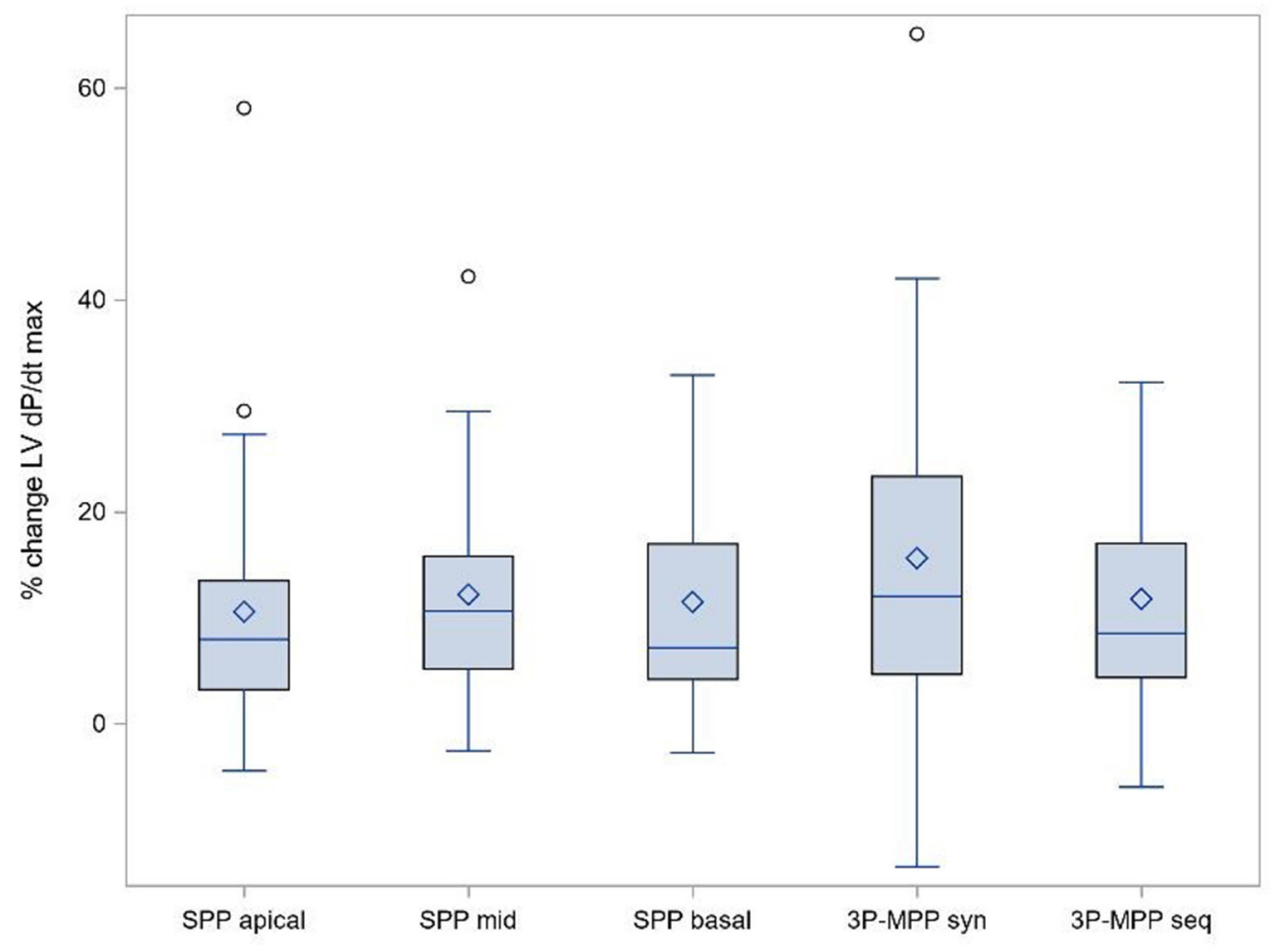
Primary objective: % ΔLV + dPdt_max_ boxplot at best AV-delay. SPP, RV-LV Single-point pacing and MPP, RV-LV Multi-point pacing. MPP_seq_, Sequential MPP; MPP_syn_, Synchronous (simultaneous) MPP; SPP_basal,mid,apical_, SPP from base, mid, apical LV electrode. Solid line depicts the median value, and boxes are 25th and 75th percentile. Whiskers represent the most extreme data point within 1.5x interquartile range from the boxes. Diamonds represent mean value, and dots are outliers.

We observed an increase in %ΔLV + dP/dt_max_ for all pacing configurations at the optimized AV delay: 3P-MPP_syn_ (15.6%, 95% CI: 8.8-22.5%), 3P-MPP_seq_ (11.8%, 95% CI: 7.6-16.0%), SPP_basal_ (11.5%, 95% CI: 7.1-15.9%), SPP_mid_ (12.2%, 95% CI: 7.9-16.5%), and SPP_apical_ (10.6%, 95% CI: 5.3-15.9%). Comparisons between 3P-MPP_syn_ and SPP configurations, 3PP-MPP_seq_ and SPP configurations as well as between 3P-MPP_syn_ and 3P-MPP_seq_ based on the weighted within-patient differences were not statistically significant except for comparison between 3P-MPP_syn_ and SPP_apical_ (3.2%, 95% CI: 0.3-6.0%, *p* = 0.03) as well as 3P-MPP_seq_ and SPP_apical_ (3.3%, 95% CI: 0.3-6.4%, *p* = 0.04) (%ΔLV + dPdt_max_ boxplot at best AV-delay is shown in Figure 3). The sensitivity analysis seemed to indicate that different results between weighted *t*-test, unweighted *t*-test and Wilcoxon signed-rank test were mainly due to the weighting of individual subjects rather than strong violation of the assumption of normality for the *t*-tests.

Fifteen patients (15/25; 60%) showed an acute hemodynamic response in at least one pacing configuration. Acute hemodynamic responder rates (i.e., AHR) varied between pacing configurations: 36% (9/25) for SPP_apical_, 44% **(**11/25) for SPP_basal_, 54% (13/24) for SPP_mid_, 56% (14/25) for 3P-MPP_syn_ and 48% (11/23) for 3P-MPP_seq._ Overall, AHR was similar for MPP configurations and SPP configurations.

### Effect of LBBB Morphology

**Figure 4:**
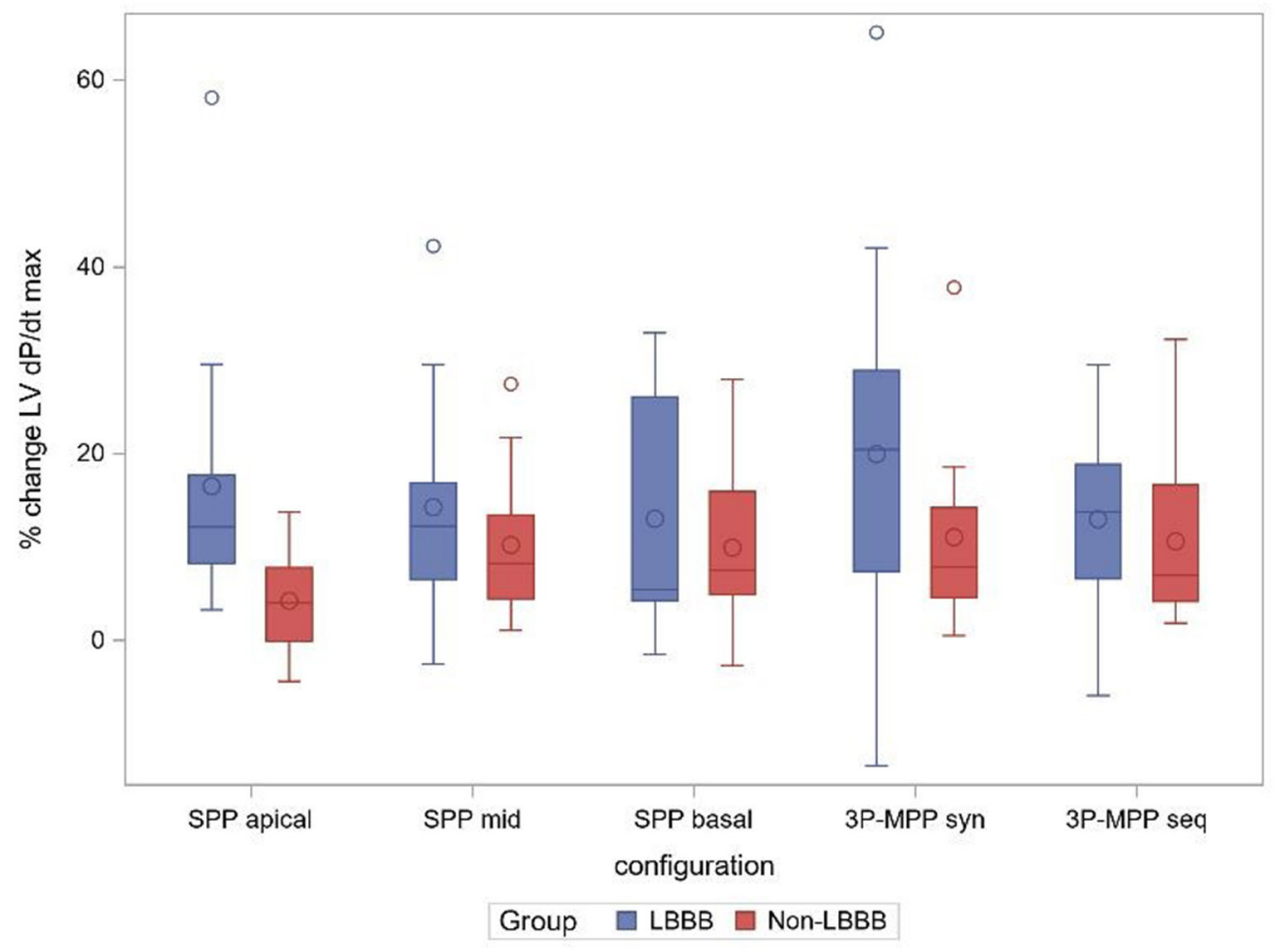
% change ΔLV + dPdt_max_ boxplot at best AV-delay in the subgroups LBBB and non-LBBB. SPP, RV-LV Single-point pacing and MPP, RV-LV Multi-point pacing. MPP_seq_, Sequential MPP; MPP_syn_, Synchronous (simultaneous) MPP; SPP_basal,mid,apical_, SPP from base, mid, apical LV electrode. Solid line depicts the median value, and boxes are 25th and 75th percentile. Whiskers represent the most extreme data point within 1.5x interquartile range from the boxes. Diamonds represent mean value, and dots are outliers.

Patients had a mean QRS-duration of 158.7 ± 11.9 ms, and 52% (13/25) of patients presented with typical LBBB pattern on ECG. As shown in Figure 4, the acute hemodynamic effect (%ΔLV + dP/dt_max_) trended higher for all pacing configuration in patients with a LBBB. The AHR in at least one pacing configuration was (77%, 10/13) for patients with a typical LBBB compared to patients with a non-LBBB (42%, 5/12) (*p* = 0.11).

### Effect of QRS Duration

Percentage change QRS duration (%ΔQRS duration) increased by 3-9% for most pacing configurations [SPP_basal_ (4.9% ± 16.5), SPP_mid_ (3.2 ± 14.9%), SPP_apical_ (8.7% ± 18.0), and MPP_seq_ (8.5% ± 19.7)], but decreased by 4.3% with 3P-MPP_syn_ (−4.3% ± 14.3). No significant correlation emerged between %ΔQRS duration and %ΔLV + dP/dt_max_ (*N* = 24, ρ = −0.28, 95% CI: −0.44-0.10).

### Effect of QLV-Delay

The Q-LV/QRS timings ranged from 0.46 ± 0.21 on the apical electrode to 0.55 ± 0.23 and 0.56 ± 0.24 on the mid and basal electrode, respectively. No significant correlation was found between the Q-LV/QRS ratio and the acute hemodynamic effect (%ΔLV + dP/dt_max_) for the whole study group with available data (*N* = 20, ρ = 0.20, 95% CI: −0.06-0.44). However, Q-LV/QRS ratio correlated more strongly with %ΔLV + dP/dt_max_ for patients with non-LBBB (N = 9, ρ = 0.41, 95% CI: 0.04-0.69), but not with LBBB. Q-LV/QRS ratio correlation with %ΔLV + dP/dt_max_ was lower (*N* = 11, ρ = 0.03, 95% CI: −0.33-0.37, *p* = 0.13) in LBBB patients for all LV electrodes.

## Discussion

The current study was designed to search for potential solutions to increase the effectiveness of CRT, in a group of patients initially at risk of non- or sub-response. Factors affecting suboptimal or even non-response phenomenon are well known and have been previously described (27). They have been listed in Supplementary Table 2. Nevertheless current expert opinions (28, 29) indicate that majority of those factors might be easily modifiable and managed by systematic and methodological algorithms of care. LV lead location and LV pacing modes and types—in case of inadequate dyssynchrony correction—remain one of main reason of non-satisfying response and are challenging.

In this acute hemodynamic study, we explored whether CRT, delivered using 3P-MPP_syn_ or 3P-MPP_seq_ is superior to SPP in patients who are likely sub-responders using low-variance measurement of the acute hemodynamic response (8, 30). Several findings have emerged. **First**, 3P-MPP_syn_ and 3P-MPP_seq_ were not superior to SPP. **Second**, a trend toward an AHR in at least one pacing configuration was observed in patients with a typical LBBB morphology, but in less than half of patients without.

### Acute Hemodynamic Response

The AHR rate for our population of patients with myocardial scar, or QRS ≤ 150 or the absence of LBBB was indeed low (~44%). This was considerably lower than the response rate of 96% (23/24) observed in the iSPOT study [in patients with CRT indication and presence of LBBB using the 4 pacing configurations and otherwise a completely comparable protocol: (8)]. Our study confirms the necessity for tailored patient selection for CRT and multipoint LV pacing as proposed by authors (15).

The range of the hemodynamic effect within an individual patient is large (data shown in Supplementary Figure 1). In this study this is especially obvious because of the small standard error for each individual patient configuration, as enforced by the specific measurement protocol applied. This allows within patient assessment, which would otherwise not be possible. In 40% of the patients we find no response (%ΔLV + dP/dt_max_ <10%) for any configuration (*consistent hemodynamic non-responders*). In 24% patients we find an acute hemodynamic response (%ΔLV + dP/dt_max_ ≥ 10%) independent of the configuration (*consistent hemodynamic responders*). And finally, in the remaining 36% patients we find an acute hemodynamic response only in some of the tested configurations. This last group is clinically the most relevant one, as choosing the right configuration will make the difference between acute hemodynamic response and non-response and thus result in reversed remodeling of LV and long-term patient benefit (31). However, identifying the LV lead position to obtain the maximal possible hemodynamic effect is beyond today's clinical practice, and new non-invasive approaches are clinically needed. QLV/QRSd was not strongly associated with acute hemodynamic response at group level (32). Optimization of the pacing configuration of CRT (with a quadripolar LV lead) is best to rely on functional assessment of cardiac function, instead of local electric delay (32).

### Multi-Point Pacing

In the present study, 3P-MPP_syn_ was the optimal configuration in 36% of all patients which was almost statistically significantly higher than the value of 20% expected by chance (one sided *p*-value = 0.03). At the same time 3P-MPP_syn_ demonstrated the highest acute hemodynamic benefit. Moreover, 3P-MPP_syn_ was the optimal configuration in 47% of those 15 patients who demonstrated an AHR in at least one configuration which was significantly higher than the proportion 21% expected by chance (one sided *p*-value *p* < 0.01). This indicates that MPP appears to consistently display better hemodynamic response.

In an pressure-volume loop study of 44 patients, Pappone et al. (6) showed that the best MPP vector configuration was associated with a greater ΔLV + dP/dt_max_, stroke work, stroke volume and LVEF, compared with the best SPP vector configuration. Thibault et al. also showed that MPP_syn_ was associated with a higher ΔLV + dP/dt_max_ than AAI pacing and that MPP was superior to SPP in 72% patients (33). These data however, maybe confounded by their experimental setup favoring positive outcomes in MPP attributed to multiple MPP configurations vs. one BiV setting using the distal electrode.

In the present study, 3P-MPP_seq_ was the optimal configuration in 28% of all patients which was not significantly higher than the value of 20% expected by chance (one sided *p* = 0.17). 3P-MPP_seq_ had similar mean AHE as SPP_mid_ and SPP_basal_. An acute hemodynamic effect emerged compared to SPP-apical, which must however be attributed to the relatively lesser effect of SPP-apical stimulation. In normal sinus rhythm, electrical impulses travel through the rapid conduction system from the His bundle toward the apex. Thereafter, LV activation spreads from apex to base as impulses exit the Purkinje system into the slower-conducting working myocardium (34). Accordingly, pacing at the apex would thus be expected to provide a physiological sequence of activation. Indeed, computer-modeling studies suggest that LV pacing guided by what is closest to normal activation is superior to pacing the latest activated region (35). In canine LBBB models, the highest hemodynamic response to CRT is observed with LV apical positions, rather than with basal and mid positions (36). This is consistent with our previous publication of a better hemodynamic response from LV apical pacing compared to basal LV pacing in patients with ischemic cardiomyopathy and a LBBB (37). Kandala et al. (38) showed that in patients with a LBBB a longer Q-LV in apically positioned LV leads was associated with more favorable LV reverse remodeling and better outcomes, compared to apically positioned LV leads with shorter Q-LV. Lercher et al. showed that a greater AHE (%ΔLV + dP/dt_max_) could be achieved by synchronizing pacing to the earliest activated segment (39). They found that the AHR (i.e., change in systolic blood pressure) was highest when pacing from with distal to basal poles. Together, these findings suggest that mimicking physiological activation by using interpole electrical separation, from apex to base, could be beneficial. In the present study, however, no advantage of 3P-MPP_seq_ was observed.

Collectively the two MPP configurations achieved the highest acute hemodynamic response in 16/25 (64%) patients which was significantly higher than the value (39.2%) expected by chance (one sided *p* < 0.01).

### LBBB Morphology

Sub-analyses of both REVERSE (40) and MADIT-CRT (41) suggested a reduced benefit in patients with non-LBBB QRS morphology. In the present study, we found that a typical LBBB morphology, even in patients with a QRS ≤ 150 ms or myocardial scar, trended toward a higher AHR (albeit small sample size in the current study). This is consistent with the importance placed on LBBB morphology by clinical guidelines (42).

According to recent studies, sequential His bundle pacing (HBP) followed by left ventricular (LV) pacing [His-Optimized CRT (HOT-CRT)] improves ventricular electrical synchrony beyond BiV and MPP (43, 44). In Vijayaraman et al. study (43) clinical response in HOT—CRT patients was also observed in CRT non-responders and non-LBBB patients. Similarly, Jastrzebski et al. (45) showed the best effect of electrical resynchronization and a higher percentage of clinical improvement in the left bundle branch area pacing—optimized CRT (LOT—CRT) group. On the other hand, Senes at al. (46) showed a better ECG effect in patients with HBP or HOT-CRT, but no clinical improvement compared to the conventional BIV pacing. However, large and randomized trials we needed.

### Electrical Evaluation

A metanalysis of individual patient data from randomized, controlled trials suggested that the survival benefit from CRT starts at a QRS > 140 ms, with less clear benefit between 120 and 140 ms (47). We found that QRS duration increased by 3-9% in most pacing protocols, with the exception of 3P-MPP_syn_, which led to a reduction. As in other studies (32, 48) we have observed no correlation between intrinsic QRS duration and ΔLV + dP/dt_max_ nor between ΔQRS duration and ΔLV + dP/dt_max_. In this study, Q-LV/QRS were lower (0.46-0.56) than observed in patients with LBBB and greater QRS durations (typically around 0.80) (8). In this respect, a low Q-LV/QRS has been shown to relate to worse clinical outcomes (48, 49).

### Clinical Implications

This study shows that even in patients with a reduced likelihood of response to, a typical LBBB morphology seems still associated with an improved acute hemodynamics. Our findings indicate that tailoring of pacing configurations (i.e., pacing electrode and optimizing the program) is required to achieve an acute hemodynamic effect in individual patients on the borderline of an clinically relevant hemodynamic response. Whilst our findings support the use of MPP as an option in some patients, it has no clear general benefit in the entire potentially predisposed group.

### Limitations

The small sample size is an important limitation, especially for the comparison between LBBB and non-LBBB patients. Therefore, other group comparisons like scar and no-scar or QRS < or > than 150 ms were not performed. The current study was a relatively small, but multicenter, non-randomized study. Furthermore, only acute hemodynamic measurements were used to define the optimal CRT device setting resulting in the best CRT-response. The results observed in this study should be tested in a larger cohort including besides acute hemodynamic measurements also longer term echocardiographic and clinical outcomes (50).

## Conclusions

No acute hemodynamic advantage emerged for 3P-MPP_syn_ or 3P-MPP_seq_ compared to SPP pacing configuration in patients with higher likelihood of CRT sub-response, except when compared to LVapical pacing.

## Data Availability Statement

The original contributions presented in the study are included in the article/Supplementary Material, further inquiries can be directed to the corresponding author.

## Ethics Statement

Study has been approved for all participating sites by Local Ethics Committees and all participants signed informed consent forms: 1. Komisja Bioetyczna Śląskiego Uniwersytetu Medycznego w Katowicach, Katowice, Poland. 2. Eticka Komisia NUSCH Pod Krasnou Horkou 1 83348 Bratislava Slovakia (Slovak Republic). 3. Comité Ético de Investigación Clínica (Instituto de Investigación Sanitaria La Fe), Valencia, Spain. 4. West Midlands - Edgbaston Research Ethics Committee The Old Chapel Royal Standard Place Nottingham NG1 6FS. 5. Regionala Etikprövningsn ämden Afdelning 1 Sandgatan 1 22350 Lund Sweden. The patients/participants provided their written informed consent to participate in this study.

## Author Contributions

All authors listed have made a substantial, direct, and intellectual contribution to the work and approved it for publication.

## Funding

The authors declare that this study received funding from Medtronic, Inc., Bakken Research Center (BRC), Maastricht, The Netherlands. The funder had the following involvement in the study: a role in study design, data collection and analysis (including statistician and database management), decision to publish, preparation of the manuscript and submission process.

## Conflict of Interest

VS, FLem, and BS were employed by the company Medtronic Inc. BM and RC were an employee of Medtronic and holds Medtronic stocks. MS, AM, AS, and FLey they received fees from commercial companies. The remaining authors declare that the research was conducted in the absence of any commercial or financial relationships that could be construed as a potential conflict of interest.

## Publisher's Note

All claims expressed in this article are solely those of the authors and do not necessarily represent those of their affiliated organizations, or those of the publisher, the editors and the reviewers. Any product that may be evaluated in this article, or claim that may be made by its manufacturer, is not guaranteed or endorsed by the publisher.
