# Supplementary material for: Acute Hemodynamic Effects of Simultaneous and Sequential Multi-Point Pacing in Heart Failure Patients With an Expected Higher Rate of Sub-response to Cardiac Resynchronization Therapy: Results of Multicenter SYNSEQ Study
Source: Front Cardiovasc Med. 2022 May 12;9:901267. doi: 10.3389/fcvm.2022.901267 (PMC9133424; doi:10.3389/fcvm.2022.901267)
Supplement: Supplementary file 1 [file Table_1.docx]

**Supplementary Table 1: Electrophysiology study duration**

| Subject characteristics | Total subjects in EP analysis cohort  (N=25) |
| --- | --- |
| EP visit duration (min.) |  |
| Mean (Standard Deviation) | 87,3 (63,7) |
| Median | 48,0 |
| 25^th^ percentile – 75^th^ Percentile | 43-149 |
| Minimum – Maximum | 35-230 |
| Number of subjects with measurements available (N,%) | 25 (100%) |
