# Supplementary material for: Acute Hemodynamic Effects of Simultaneous and Sequential Multi-Point Pacing in Heart Failure Patients With an Expected Higher Rate of Sub-response to Cardiac Resynchronization Therapy: Results of Multicenter SYNSEQ Study
Source: Front Cardiovasc Med. 2022 May 12;9:901267. doi: 10.3389/fcvm.2022.901267 (PMC9133424; doi:10.3389/fcvm.2022.901267)
Supplement: Supplementary file 2 [file Table_2.docx]

**Supplementary Table 2.** Most important and commonly met factors affecting sub- or non-response to CRT.

| A-V delays programming unadjusted to native atrio-ventricular conduction |
| --- |
| BiV pacing <90-95%  Concomitant arrhythmias (i.e AFib, PVCs) |
| Suboptimal heart failure pharmacotherapy |
| **LV lead location/persistent dyssynchrony** |
| Inadequate qualification/off-label CRT-use |
| Choice of CRT-P vs CRT-D |
| Concomitant diseases |
| Patient-related factors (compliance, recommendation adherence) |

Factor related to LV lead location and LV pacing performance (bolded) is directly related to presented study results.
